# Process Development for Benzyl Alcohol Production by Whole-Cell Biocatalysis in Stirred and Packed Bed Reactors

**DOI:** 10.3390/microorganisms10050966

**Published:** 2022-05-03

**Authors:** Carlos J. C. Rodrigues, Carla C. C. R. de Carvalho

**Affiliations:** 1 Department of Bioengineering, iBB—Institute for Bioengineering and Biosciences, Instituto Superior Técnico, Universidade de Lisboa, Av. Rovisco Pais, 1049-001 Lisbon, Portugal; carlos.junio@tecnico.ulisboa.pt; 2 Associate Laboratory i4HB—Institute for Health and Bioeconomy, Instituto Superior Técnico, Universidade de Lisboa, Av. Rovisco Pais, 1049-001 Lisbon, Portugal

**Keywords:** biocatalysis, two-phase biocatalysis, stirred tank reactor, packed bed reactor, whole cells, immobilization, benzaldehyde, benzyl alcohol, marine biocatalyst

## Abstract

The ocean is an excellent source for new biocatalysts due to the tremendous genetic diversity of marine microorganisms, and it may contribute to the development of sustainable industrial processes. A marine bacterium was isolated and selected for the conversion of benzaldehyde to benzyl alcohol, which is an important chemical employed as a precursor for producing esters for cosmetics and other industries. Enzymatic production routes are of interest for sustainable processes. To overcome benzaldehyde low water solubility, DMSO was used as a biocompatible cosolvent up to a concentration of 10% (*v*/*v*). A two-phase system with *n*-hexane, *n*-heptane, or *n*-hexadecane as organic phase allowed at least a 44% higher relative conversion of benzaldehyde than the aqueous system, and allowed higher initial substrate concentrations. Cell performance decreased with increasing product concentration but immobilization of cells in alginate improved four-fold the robustness of the biocatalyst: free and immobilized cells were inhibited at concentrations of benzyl alcohol of 5 and 20 mM, respectively. Scaling up to a 100 mL stirred reactor, using a fed-batch approach, enabled a 1.5-fold increase in benzyl alcohol productivity when compared with batch mode. However, product accumulation in the reactor hindered the conversion. The use of a continuous flow reactor packed with immobilized cells enabled a 9.5-fold increase in productivity when compared with the fed-batch stirred reactor system.

## 1. Introduction

Benzyl alcohol is an important aromatic alcohol used as a solvent in inks, paints, glues, and resins [1], in household cleaners and detergents [2], and as a food additive [3]. It is a member of the fragrance structural group aryl alkyl alcohols, being frequently used as a fragrance ingredient in several consumer products such as shampoos, soaps, and cosmetic products [2]. Benzyl alcohol has bacteriostatic and antiseptic properties with modest toxicity, which increases their versatility [2,4]. Benzyl alcohol has also been used as a substrate for the synthesis of esters applied as important building blocks for bulk and commodity chemicals used in pharmaceutical, fragrance, and food industries [5,6].

Industrial production of benzyl alcohol is traditionally made by hydrolysis of benzyl chloride or hydrogenation of benzaldehyde [7]. These processes use non-renewable substrates, metal catalysts, high temperatures, and high pressures, and produce by-products with deleterious environmental effects. Microorganisms may also be used for the production of benzyl alcohol. The conversion of benzaldehyde to benzyl alcohol was undertaken in 1983 by the fungus *Rhodotorula muciluginosa* immobilized in an ultrafiltration cell [8]. The conversion involved the NADP-oxidoreductase enzyme present in the cells and was used as an example of the detoxication of industrial wastewaters since benzaldehyde commonly occurs in wastewaters produced by the pharmaceutical and cosmetic industries. More recently, Pugh et al. proposed the *de novo* production of benzyl alcohol with engineered microorganisms from renewable glucose [9]. The engineered route included a step for the reduction of benzaldehyde to benzyl alcohol by the combined activity and native regulation of multiple endogenous alcohol dehydrogenases and/or aldo-ketoreductases. Final benzyl alcohol titers of ca. 114 mg/L, at a yield of 7.6 mg/g glucose, were attained. Oba et al. produced benzyl alcohol using vegetable wastes such as bean pods, green apple peel and avocado seeds as the source of enzymes [10]. The highest conversions of benzaldehyde were obtained with the capulin (86%) and mamey seeds (77%). Other studies have reported the production of benzyl alcohol by fermentation: the yeast *Hanseniaspora vineae* was found able to produce it by *de novo* synthesis in the absence of grape-derived precursors during wine fermentation [11]; an artificial enzyme cascade was developed in *Escherichia coli* to produce benzyl alcohol directly from L-phenylalanine [12]. However, the low amounts produced in these processes still render their implementation unfeasible. Therefore, solutions for a sustainable large-scale production of benzyl alcohol are needed.

Biocatalysis uses whole-cells or enzymes to increase the rate of chemical reactions with remarkable selectivity. Biocatalysts are produced from renewable resources, are biodegradable, non-hazardous and nontoxic, and work under mild conditions, which makes the processes more sustainable compared with organic chemical synthesis [13,14,15]. In recent years, the advantages of biocatalysts and the request for more sustainable processes pushed forward the implementation of biocatalysts for production of valuable molecules at the industrial level [16,17,18]. New biocatalysts are needed for different applications to fulfill the needs of our society [19]. Marine environments are an important source for the discovery of new biocatalysts [20,21]. The cultivation of microorganisms in the laboratory and the screening for enzyme activities are important for the discovery of novel enzymes because the approach does not rely on the comparison with protein sequence information deposited in databases of known enzymes [17,22]. In addition, this classic approach has proved to be efficient and able to be used in high-throughput systems to accelerate the discovery of new enzymes [23,24].

After the selection of the suitable enzyme for a given bioprocess, enzyme formulation is an important parameter of a reaction [25]. Whole cells, contained inside the active enzyme, are an economic catalyst formulation [26,27]. Since cellular disruption and purification steps of enzymes are not required, the cost of whole cell biocatalysts could be ca. 10-fold cheaper than that of purified enzymes [27]. For applications at industrial scale, the saving in time and costs could be significant. The negative effects on the enzymes of shear forces and organic solvents used in catalytic processes are reduced when employing whole cells [28,29]. The cell membrane serves as a protection wall between the outside environment and the enzymes inside the cells [17,30]. Additionally, whole cells are able to perform multi-step microbial bioconversions and co-factor recycling [31,32]. The conversion of benzaldehyde to benzyl alcohol in microbial cells may be catalyzed by aldehyde dehydrogenases (ALDHs), alcohol dehydrogenases (ADHs), and/or aldo-keto reductases, which are enzymes able to catalyze the reversible reduction of aldehydes, ketones, and/or keto esters to the corresponding alcohol with consumption of NADH or NADPH [33,34,35].

In this work, the production of benzyl alcohol by a marine bacterium was studied. The use of whole cells allows the natural recycling of the co-factors. Reaction conditions, reaction media composition, and operation mode in small-scale bioreactors were investigated using resting whole cells as a biocatalyst. Small scale experiments using a fed-batch stirred reactor and a continuous plug flow reactor (CPFR) indicated the potential of the studied system for future applications. Our study demonstrates how screening for suitable marine biocatalysts and reaction conditions, together with the solving of bioengineering problems, may lead to bioprocesses for the production of benzyl alcohol at the g/L scale. This may help, in the near future, in the development of an industrial sustainable process using biotechnological solutions.

## 2. Materials and Methods

### 2.1. Biocatalyst Isolation and Identification

Strain 232 was isolated from a sample collected at Ponta do Castelo, São Miguel Island, the Azores, Portugal (37°53′27″ N, 25°49′33″ W). Sampling conditions have been described previously [21]. Briefly, the temperature of the water in the intertidal pool was 19 °C, the air temperature was at 17 °C, the pH was 9.25, and the conductivity of the water was 96.1 mS/cm. The strain was isolated in the laboratory in tryptic soy agar (Becton Dickinson GmbH, Heidelberg, Germany) diluted 1:100.

The extraction of DNA from the colonies was carried out using the DNeasy Powerwater kit (Qiagen GmbH, Hilden, Germany). DNA purification, PCR amplification of a segment of the 16S rRNA gene containing the variable regions V1-V9, and alignment of sequences to generate the consensus sequence were conducted by Stab Vida (Lisbon, Portugal). BLAST analysis of the consensus sequence was undertaken at the National Center for Biotechnology Information website (https://blast.ncbi.nlm.nih.gov/Blast.cgi; accessed on 28 June 2021 and repeated on 1 March 2022). The isolate presented a 100% similarity to sequences of several *Glutamicibacter arilaitensis* isolates listed (including *G. arilaitensis* Re117 and several strains isolated from the surfaces of cheeses), and are referred as *G. arilaitensis* 232 in the remainder of the text.

*G. arilaitensis* 232 cells were identified as being able to convert benzaldehyde to benzyl alcohol at interesting levels during a high throughput screening of our library of marine bacteria using aldehydes and ketones as substrates.

### 2.2. Biocatalyst Preparation

*G. arilaitensis* 232 cultures were grown in 2 L Erlenmeyer flasks containing 400 mL of tryptic soy broth (TSB; Becton, Dickinson and Company, Franklin Lakes, NJ, USA). The cultures were grown overnight at 30 °C and 180 rpm in an orbital incubator (Agitorb 200, Aralab, Rio de Mouro, Portugal). Cells were harvested from the liquid broth by centrifugation in 500 mL centrifuge tubes at 7000 rpm and 4 °C (Sorvall^®^ RC 6 centrifuge from Thermo Scientific^TM^, Waltham, MA, USA), and washed with 100 mM Tris-HCl buffer pH 7.5. The cells were stored at 4 °C until utilization. Whole cells were used in the bioconversion assays as free cells or immobilized. Immobilization in sodium alginate was used by occlusion of whole cells [36,37]. The immobilization was carried out as follows: 1 g of sodium alginate was dissolved in 30 mL of distilled water at 45 °C; after cooling, 5 mL of cell suspension was added to the sodium alginate mixture; alginate beads containing the cells were produced using a 1000 µL pipette by extrusion dripping to a stirred solution of calcium chloride (2%, *w*/*v*). The spheres formed were collected and rinsed with a washing buffer (20 mM sodium acetate pH 4.5 containing 1% (*w/v*) of calcium chloride). Excess liquid was carefully removed from the calcium-alginate beads containing the cells, which were stored at 4 °C if not immediately used.

### 2.3. Single Aqueous Phase Systems

Bioconversion assays were conducted in 10 mL Verex^TM^ Headspace vials closed with screwed Verex Caps with bonded-in PTFE/silicone septa (Phenomenex, Torrance, CA, USA). Media were stirred using magnetic agitation (12 mm × 3 mm stirrer bar from Kartell, New York, NY, USA) by a Variomag^®^ Multipoint 15 Magnetic Stirrer system with a Telemodul 20 C external controller (Thermo Scientific, Waltham, MA, USA) which was placed inside an incubator (Certomat^®^ H from B. Braun, Melsungen, Germany) at 30 °C.

#### 2.3.1. Aldehyde and Ketone Screening

The conversions of the aldehyde benzaldehyde (from Fisher Scientific, Thermo Fisher Scientific Inc, Waltham, MA, USA), and of the ketones 2-acetylpyridine, α-tetralone, 4′-(trifluoromethyl)acetophenone, isobutyrophenone, 1-indanone, 2-acetylpyraine, cyclohexyl phenyl ketone, 4′-methoxyacetophenone, and benzophenone (all from Sigma-Aldrich, St. Louis, MO, USA) were performed in triplicate. The reaction medium had a final volume of 1.2 mL and contained 10% (*v*/*v*) of dimethyl sulfoxide (DMSO), whole *G. arilaitensis* 232 cells at 10.42 mg mL^−1^ of dry cell weight (DCW), and 5 mM of the respective ketone or aldehyde tested in 100 mM Tris-HCl pH 7.5 buffer. The medium was stirred at 800 rpm. Samples collected at the start (0 h) and after 24 h of reaction were collected and processed for gas chromatography-mass spectrometry (GC-MS) analysis for determination of substrate conversion and product formation.

#### 2.3.2. Effect of Cosolvent and Substrate Concentrations

Reactions for cosolvent selection contained: 10% (*v*/*v*) of the tested cosolvent (DMSO, methanol, acetone, acetonitrile, or *iso*-propanol), 10 mg mL^−1^ of DCW of biocatalyst, and 20 mM benzaldehyde in 100 mM Tris-HCl pH 7.5 buffer. The assays with 1 mL volume and stirred at 800 rpm were performed in triplicate. Bioconversions to determine the optimal concentration of DMSO were carried out under the same conditions but with different percentages (0–50%) of DMSO in the reaction vial. The reaction was stopped after 2 h of reaction when 500 µL of ethyl acetate was added for extraction of substrate and product, which were analyzed by GC-MS.

To assess the effect of the initial concentration of substrate on *G. arilaitensis* 232 activity, the reaction mixture contained: 5% (*v*/*v*) of DMSO, 12.6 mg mL^−1^ of DCW, 5 to 75 mM of benzaldehyde in 100 mM Tris-HCl pH 7.5 buffer. Different samples were collected during the first 90 min of the reaction and processed for GC-MS analysis. The quantification of benzyl alcohol was used for the calculation of relative activity. The 1 mL assays were performed at 30 °C and 800 rpm, in triplicate.

### 2.4. Two-Phase Systems

The effects of using an organic solvent as substrate reservoir and of the stirring speed on the bioconversion of benzaldehyde to benzyl alcohol were assessed in a two-phase system. The aqueous phase (1 mL) contained: 5% (*v*/*v*) of DMSO and 10.9 mg mL^−1^ of DCW in 100 mM Tris-HCl pH 7.5 buffer. The organic phase (1 mL) contained: 50 mM of benzaldehyde dissolved in *n*-hexane, *n*-heptane, or *n*-hexadecane. Each organic solvent was tested in the bioreactor magnetically stirred at 200, 400, 800, and 1000 rpm. The assays were performed in triplicate at 30 °C. After 1 h of conversion, 150 µL of each phase were collected from the bioreactor to separate microtubes (Eppendorf, Hamburg, Germany), and processed for GC-MS analysis.

To assess the effect of benzaldehyde concentration in biocatalyst performance in the two-phase systems, the following media were used: an aqueous phase (2 mL) containing 5% (*v*/*v*) of DMSO and 62.3 mg mL^−1^ of DCW in 100 mM Tris-HCl pH 7.5 buffer; and an organic phase (2 mL) containing benzaldehyde at different concentrations (up to 500 mM) in *n*-hexane. Samples of the organic phase were collected at 0, 20, and 40 h of reaction and analyzed by GC-MS. Reactions were performed at 200 rpm and 30 °C, in triplicate.

### 2.5. Bioreactions with Immobilized Cells

The two-phase system was prepared with 1 mL of aqueous phase (100 mM Tris-HCl pH 7.5 buffer) and 1 mL organic solvent phase (*n*-hexane with 50 mM of benzaldehyde). The system contained 10.0 mg mL^−1^ of DCW immobilized in alginate. The effect of cosolvent was assessed by adding 5% (*v*/*v*) of DMSO in 100 mM Tris-HCl pH 7.5 buffer containing 50 mM benzaldehyde. After 2 h of reaction, samples from both phases were collected and processed for GC-MS analysis to determine benzaldehyde and benzyl alcohol concentrations.

The effect of substrate concentration on immobilized biocatalyst performance was determined in a two-phase system containing: 2 mL of aqueous phase with 5% (*v*/*v*) of DMSO, 12.0 mg mL^−1^ of DCW immobilized cells in alginate, and 100 mM Tris-HCl pH 7.5 buffer; 2 mL of organic phase containing *n*-hexane with 50, 100, or 250 mM of benzaldehyde. The concentration of benzyl alcohol was determined by GC-MS at the end of the reaction (20 h).

The product inhibition assay was performed using an aqueous system containing 5% (*v*/*v*) of DMSO. The 1 mL reaction system contained 10 mM of benzaldehyde and 12.0 mg mL^−1^ of DCW of immobilized cells in alginate in 100 mM Tris-HCl pH 7.5 buffer. Initial concentrations of benzyl alcohol (0, 5, 20, 50, and 100 mM) were added to the reaction media. After 2 h, a sample was collected and processed for GC-MS analysis.

A control assay was carried out for all experiments with the same amount of free cells. All assays, with free and immobilized cells, were carried out at 200 rpm and 30 °C, in triplicate.

### 2.6. Scale-Up of the Reaction System

#### 2.6.1. Stirred Reactor

The two-phase system was prepared in a 100 mL Duran^®^ laboratory bottle, with perforated screw cap GL 45 (Duran, DWK Life Sciences GmbH, Mainz, Germany). The 30 mL aqueous phase was composed of 5% (*v*/*v*) of DMSO and 41.35 mg mL^−1^ of DCW in 100 mM Tris-HCl pH 7.5 buffer. The 20 mL organic phase contained 50 mM of benzaldehyde in *n*-hexane. The system was operated in fed-batch mode, with benzaldehyde being added to the bioreactor after its depletion in the reaction media. The vessel was maintained inside an incubator (Certomat^®^ H from B. Braun, Melsungen, Germany) at 30 °C and was stirred at 135 rpm (IKA^®^ RW 11 “Lab egg” stirrer, Staufen, Germany). Samples (100 µL) from both phases were collected over time. Aqueous samples were extracted with 100 µL of ethyl acetate and organic samples were used directly for analysis. Product and substrate were quantified by GC-MS analysis.

#### 2.6.2. Plug Flow Reactor

A plug flow reactor with immobilized cells in sodium alginate was tested. A jacked glass column with 25 mL of volume was packed with 19.2 g of immobilized whole cells corresponding to 273.55 mg of DCW. The volume of liquid inside the column with the biocatalyst was 7 mL. A peristaltic pump (205S from Watson Marlow, Falmouth, UK) was used to pump the substrate solution, containing 5% (*v*/*v*) DMSO and 15 mM benzaldehyde in 100 mM Tris-HCl pH7.5 buffer, from a reservoir to the inlet at the top of the reactor column. The residence time inside the bioreactor was 1.5 h. The bioreactor was maintained at 30 °C by passing water on the column jacket using a water bath (Corio CD from Julabo GmbH, Seelbach, Germany). Samples were taken over time to monitor substrate and product concentrations by GC-MS analysis.

### 2.7. Analytical Methods

The collected aqueous samples were immediately extracted with ethyl acetate (at 1:1 (*v*/*v*), if not stated otherwise). The ethyl acetate layer was extracted and dried with magnesium sulphate, followed by centrifugation for 1 min at 10,000 rpm (microcentrifuge μSpeedFuge^®^ SFA13K from Savant, Hyannis, MA, USA). Clear ethyl acetate containing the analytes was placed in GC vials before analysis. Organic samples from the two-phase systems were dried with magnesium sulphate and centrifuged as mentioned for the aqueous samples and added to GC vials. The substrate and product concentrations were determined by GC-MS. The equipment used was an Agilent 7820A GC equipped with a 7693A autoinjector, and an Agilent 5977E quadrupole MS detector (all from Agilent Technologies, Santa Clara, CA, USA). The capillary column used was an Agilent J&W Ultra-2, working at a constant flow of 1 mL min^−1^. The GC injector was set at 200 °C, the MS source at 230 °C, the MS quad at 150 °C, and the MSD transfer line at 280 °C. The separation of substrates and products was achieved by programming the oven to an initial temperature of 40 °C, and increasing the temperature to 240 °C at 38 °C min^−1^. Peak identification was carried out by comparison of MS data with those of injected standards using the software Qualitative Analysis, whilst peak quantification was done using Quantitative Analysis, both part of the MassHunter Workstation from Agilent.

### 2.8. Statistical Analysis

Statistical analysis was performed using Microsoft^®^ Excel (Albuquerque, NM, USA). Significant differences between activities were determined by one-way ANOVA. A *p* value < 0.05 was deemed significant.

## 3. Results and Discussion

### 3.1. Screening for Activity with Aromatic Ketones

**Figure 1:**
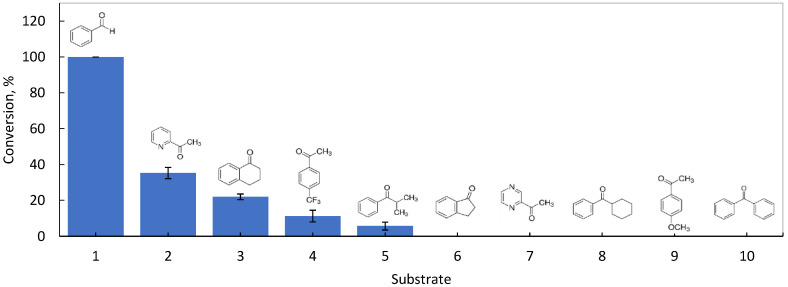
Conversion at 24 h of an aldehyde and nine different ketones by the marine bacterium *G. arilaitensis* 232. 1. benzaldehyde; 2. 2-acetylpyridine; 3. α-tetralone; 4. 4′-(trifluoromethyl)acetophenone; 5. isobutyrophenone; 6. 1-indanone; 7. 2-acetylpyrazine; 8. cyclohexyl phenyl ketone; 9. 4′methoxyacetophenone; and, 10. benzophenone. Results are the average of three independent bioconversions. Data are presented as mean ± standard deviation.

The production of alcohols from the respective ketones or aldehydes is interesting for the production of more complex molecules as intermediates for several industries [18,38,39]. In the present study, the ability of the bacterium *G. arilaitensis* 232, isolated from a marine sample collected in the Azores, Portugal, for the conversion of aromatic ketones/aldehydes, was tested using nine ketones and one aldehyde (Figure 1). The results showed conversion for half of the substrates tested. The complete conversion only occurred with the substrate benzaldehyde. The second most converted substrate was 2-acetylpyridine (35.3%), followed by α-tetralone (22.0%), 4′-(trifluoromethyl)acetophenone (11.3%) and isobutyrophenone (5.7%). A search in the KEGG BRITE Database [40] indicates the presence of at least one benzaldehyde dehydrogenase (NADP^+^ dependent), three aldehyde dehydrogenases (NAD^+^ or NADP^+^ dependent), and at least two alcohol dehydrogenases (NADP^+^ dependent) in *G. arilaitensis.* This bacterial species has been found to be associated with late stages of cheese ripening and identified as being responsible for the production of alcohols, carboxylic acids, and ketones [41,42]. The genus *Glutamicibacter* belongs to the phylum Actinomycetota. The marine actinomycete diversity and their ability to produce secondary metabolites and interesting enzymes for biotechnology are largely acknowledged [7,43]. Benzaldehyde derivatives have been found to be produced in marine actinomycetes isolated from seaweed [44]. Highly volatile solid and carbon content analysis of seaweeds showed the presence of, among other compounds, aldehydes and ketones [14], while aldehydes and ketones have been found to be photoproducts from solar-irradiated crude oil-seawater [45], and to be part of the volatile organic compounds (VOCs) in seawater [46]. The presence of aldehydes and ketones in seawater may contribute to the presence of active enzymes in the isolated bacterium.

The conversion of aromatic aldehydes and aromatic-aliphatic ketones such as acetophenones and acetylpyridines is not, to our knowledge, common in a single biocatalyst. Nevertheless, the filamentous fungus *Didymosphaeria igniaria* KCH 6670 was reported to carry out the asymmetric reductions of prochiral aromatic-aliphatic ketones, including acetonaphthones, acetophenones, and acetylpyridines, to mainly the corresponding (*S*)-alcohols [47]. In the present study, the cells converted nonetheless 2.8-fold more benzaldehyde than any of the other substrates (Figure 1). The product, benzyl alcohol, besides being the smallest molecule tested, is able to fluidize the cellular membrane of bacterial cells [48,49], which could have helped the mass transfer of the substrate and product in the bioconversion. The conversion of benzaldehyde to benzyl alcohol (Scheme S1 in Supplementary Information) was chosen for further process development studies using the *G. arilaitensis* isolate 232 cells.

### 3.2. Bioprocess Development

#### 3.2.1. One-Phase System

Biocatalytic reactions may occur in diverse media, from the classic aqueous systems, with a pure aqueous environment, to unconventional systems using organic solvents, ionic liquids, and supercritical fluids with low water content [17,26,29,50]. The choice of the system depends on various factors, such as substrate solubility, enzyme stability, and/or downstream processing, and normally requires experimental work to adjust the media to biocatalyst performance [51].

**Figure 2:**
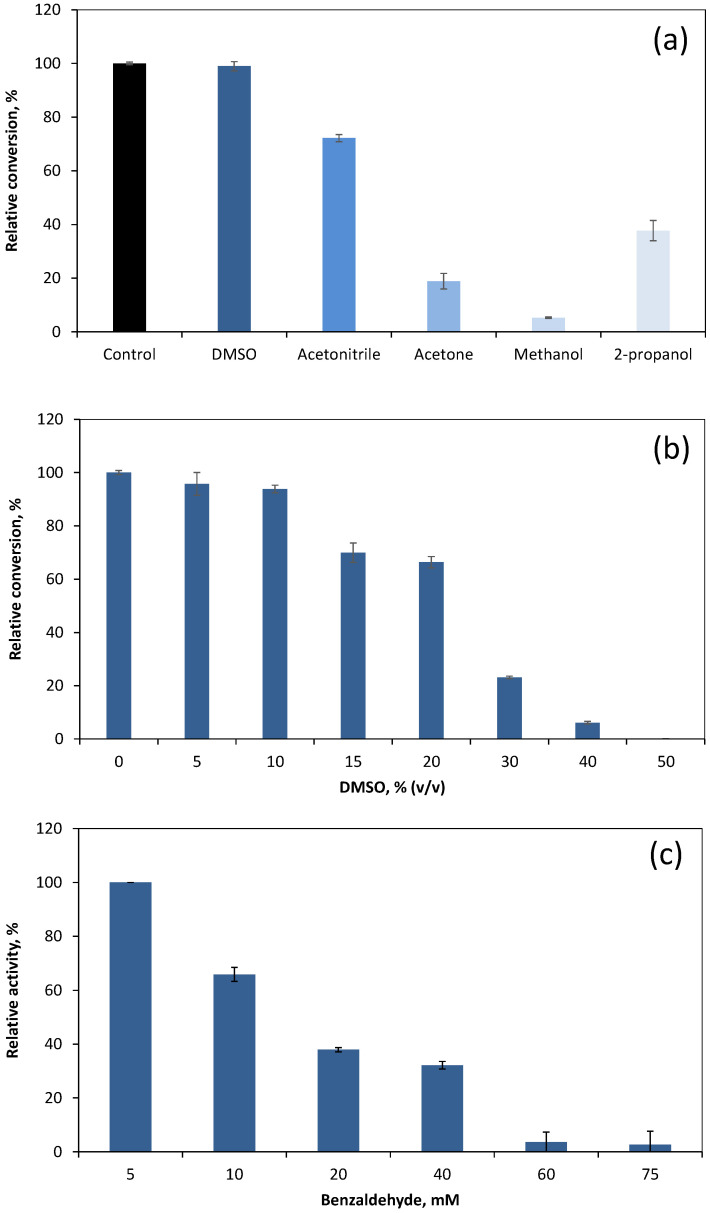
Relative conversion of benzaldehyde to benzyl alcohol by *G. arilaitensis* 232 cells after 2 h in an aqueous system with a cosolvent. (**a**) Conversion of benzaldehyde in the presence of different organic solvents used as cosolvent in relation to a control without organic solvent. (**b**) Influence of DMSO concentration on the conversion of benzaldehyde relative to a system without DMSO. (**c**) Effect of benzaldehyde concentration on the activity of the biocatalyst, relative to a system with 5 mM benzaldehyde. Results are the average of three independent bioconversions. Data are presented as mean ± standard deviation.

The low water solubility of benzaldehyde (65.49 mM) requires the addition of a cosolvent to an aqueous system to increase its solubility and diffusion if an industrial application is envisaged. To assess the effect of solvents used as cosolvents on biocatalyst performance, DMSO, acetonitrile, acetone, methanol, and 2-propanol were added to the bioreaction (Figure 2a). The highest conversion was achieved with DMSO, with only 1% of reduction in conversion being observed when compared with the control reaction without cosolvent added. In a previous study with a marine ω-transaminase, we also observed that DMSO was the cosolvent allowing the highest retention of activity [52]. DMSO is often mentioned as a cosolvent in the literature due to improved substrate solubility and enzyme properties, although it may difficult to extract the components from the aqueous phase and wastewater treatment [51]. Organic solvents may cause molecular and phase toxicity [53]. DMSO has a low log *p* value (−1.35, which predicts a high toxicity), but it usually does not affect whole cell biocatalysts as predicted [54]; however, it may enhance the permeability of lipid membranes [55]. This may explain the decrease in conversion observed with increasing percentage of DMSO used in the reaction (Figure 2b). Up to 10% (*v*/*v*) of DMSO, the relative conversion of benzaldehyde was 93.8% of the control without cosolvent, but a significant inhibition of biocatalyst activity was observed for concentrations of 15% (*v*/*v*) and above. The largest DMSO percentage used with detectable conversion was 40% (*v*/*v*), which reduced the biocatalyst conversion performance by 93.9%. Another possible explanation may be related to the solubility of compounds in DMSO, which is very dependent on the water:DMSO ratio because of the highly structured H-bonding network formed [56].

After selecting a concentration of 10% DMSO, the optimal pH and temperature for the benzaldehyde reduction were determined. The experiments showed the highest conversion values at 30 °C and pH 7–7.5 with Tris-HCl buffer and pH 7–8 with citrate-phosphate buffer (Figure S1). For further development, Tris-HCl pH 7.5 was selected as buffer.

The initial concentration of substrate was also found to considerably affect the conversion of benzaldehyde into benzyl alcohol (Figure 2c). In a system with 10% (*v*/*v*) DMSO and 10 mM benzaldehyde, *G. arilaitensis* 232 cells converted 65.9% of the benzaldehyde that was converted when the initial substrate concentration was 5 mM. However, the value for the relative conversion decreased to 38.0% and 3.6% when the initial concentration of benzaldehyde was 20 and 60 mM, respectively. This indicates that benzaldehyde causes substrate inhibition to the biocatalyst. Benzaldehyde is known for its toxicity [57] and inhibition of the whole cell biocatalyst was expected. Jakoblinnert and Rother also described benzaldehyde inhibition to *Pseudomonas fluorescens* cells during the implementation of a two-step biocatalytic cascade for the production of 1-phenylpropane-1,2-diol using whole cells [58].

#### 3.2.2. Two-Phase System

High concentrations of substrate(s) are desired for industrial applications of bioprocesses to assure economically viability. However, the low solubility of industrially interesting substrates hampers, in most cases, their direct application in conventional aqueous media. This may be overcome by the addition of an organic phase, which acts as a substrate and/or product reservoir [59,60].

In the present study, to increase the potential application of the reaction process, a biphasic system was tested using an immiscible organic solvent as a reservoir for benzaldehyde. This allows an increase in the concentration of benzaldehyde in the system, but, at the same time, protects the whole cell biocatalyst from the inhibitory effect of the substrate since benzaldehyde is partitioned mainly to the organic phase. The low concentrations of benzaldehyde in the aqueous phase, and thus in contact with the biocatalyst, prevent enzyme inhibition.

The selection of an organic solvent can be based on different criteria, including substrate/product solubility, toxicity towards biocatalyst, health and environmental hazards, and price [26,54,60]. We selected *n*-hexane, *n*-heptane, and *n*-hexadecane to assess the efficiency of the two-phase system. *n*-Hexane is one of the solvents commonly used in the industry with several production processes surpassing 100 kg, although it is a solvent which raises several concerns regarding environmental, health, and safety issues [60,61]. *n*-Heptane is also commonly used in, e.g., the pharmaceutical industry and is preferable in terms of safety and health concerns when compared to *n*-hexane [60,62]. *n*-Hexadecane presents a log *P* above 8 and is the most biocompatible and ‘green’ of the organic solvents tested [60], although its high price may hamper its application in industrial processes.

**Figure 3:**
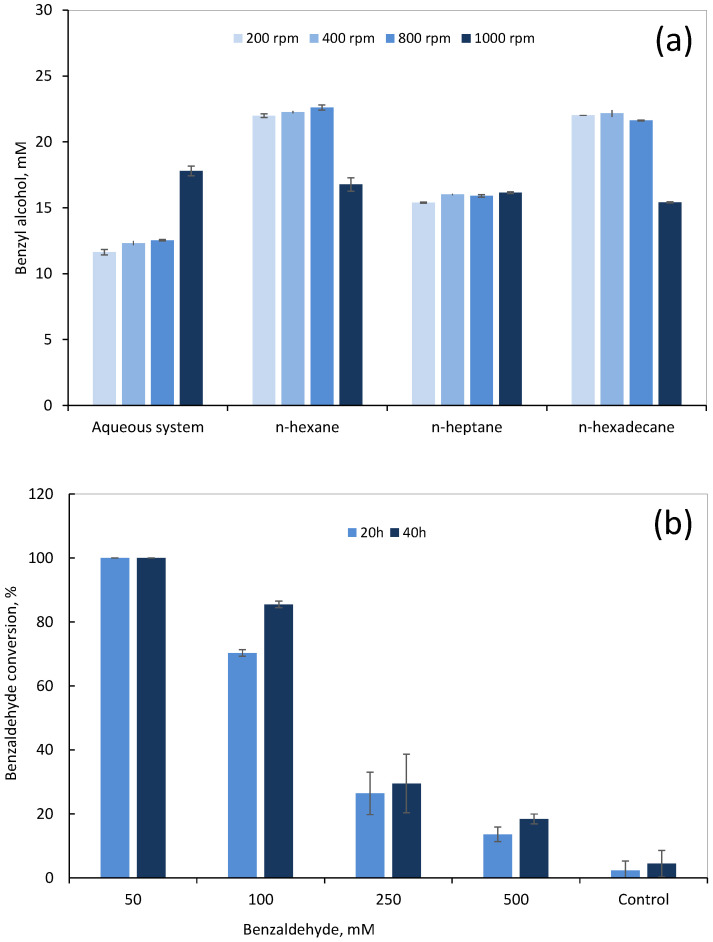
Conversion of benzaldehyde to benzyl alcohol by *G. arilaitensis* 232 cells in two-phase systems. (**a**) Production of benzyl alcohol using 3 different water immiscible organic solvents as compared with the aqueous system, at different stirring speeds (200 to 1000 rpm). (**b**) Effect of the initial benzaldehyde concentration on benzaldehyde conversion at 20 and 40 h using the two-phase system with *n*-hexane as organic solvent. Control assays were carried out without cells and 100 mM of benzaldehyde. Results are the average of three independent bioconversions. Data are presented as mean ± standard deviation.

The results of the two-phase systems were, in general, better than the aqueous phase system allowing higher concentrations of benzyl alcohol, except when the system was stirred at 1000 rpm (Figure 3a). At this high stirring speed, cell rupture may occur and the enzymes may leak into the aqueous solution containing the substrate. However, the organic phase may affect the unprotected enzymes, while causing interfacial phenomena, resulting in a lower activity [63]. Up to 800 rpm, the concentration of product was on average 1.8-fold higher when *n*-hexane (*p* = 0.01) or *n*-hexadecane (*p* = 0.02) were used as organic phase, in comparison with the single aqueous system. In the system with *n*-heptane, cells produced 1.3-fold more benzyl alcohol than the aqueous system and ca. 70% less than the other two-phase systems. It was previously observed that *n*-heptane (and *n*-octane) may induce larger changes in bacterial membranes than *n*-hexane and *n*-hexadecane, causing, e.g., increased degree of saturation and lower net surface charges [64], which may influence substrate uptake across the membrane. In *n*-hexane, the organic:aqueous partition of benzaldehyde in percentage terms was 76.7:23.3 (%:%) and a decrease with increasing number of carbon atoms in the alkanes tested was observed: to 75.1:24.9 in *n*-heptane and 66.6:33.4 in *n*-hexadecane (data not shown). Since *n*-hexane was an efficient reservoir for benzaldehyde, and allowed the highest production of benzyl alcohol, similar to that of *n*-hexadecane, but has a much lower price, *n*-hexane was selected as organic solvent to further improve the system.

The effect of different initial concentrations of benzaldehyde on cell performance was then tested using *n*-hexane as organic phase (Figure 3b). The full conversion of 50 mM benzaldehyde was observed at 20 h of reaction using the two-phase system. During the same period, cells given an initial concentration of 100 mM converted 70% of the initial benzaldehyde. At 40 h of reaction, 85% of the initial benzaldehyde was converted. However, a clear reduction in the amount of converted benzaldehyde occurred at higher concentrations of substrate: second degree polynomial trendlines could be adjusted to the curves of benzaldehyde conversion vs. benzaldehyde concentration at 20 and 40 h with *R*^2^ of 0.995 and 0.991, respectively (Figure S2). Only ca. 29% and 18% of benzaldehyde could be converted after 40h when, respectively, 250 and 500 mM benzaldehyde were initially added. Nevertheless, the *n*-hexane:aqueous phase system allowed an increased load of substrate while decreasing its inhibitory effect, which resulted in higher product concentrations. The cells were able to produce ca. 92 mM (corresponding to 9.9 g/L) of benzyl alcohol for an initial benzaldehyde concentration of 500 mM. An engineered *E. coli* containing a non-natural pathway to produce benzyl alcohol from glucose could produce 114 mg/L (1.05 mM) [9].

#### 3.2.3. Immobilized vs. Free Cells

The immobilization of whole cells is well documented and several applications at the industrial level may be found. The most successful is probably the production of acrylamide from acrylonitrile using immobilized *Rhodococcus rhodochrous* J1 [17,65]. Immobilization protects the cells from the reaction conditions, which may increase process stability and lifetime, and help product recovery [66]. Moreover, higher cell densities and specific productivities, biocatalyst reuse, and continuous bioreactors without cell wash-out are allowed. Among the immobilization methods used in industrial bioprocesses, calcium alginate is one of the most successful due to the mild gelling properties and non-toxicity, although substrate mass transfer limitations may occur [66,67]. The immobilization process by cell entrapment is simple, cheap, and well established, and the natural polymer is biocompatible and biodegradable [68,69].

**Figure 4:**
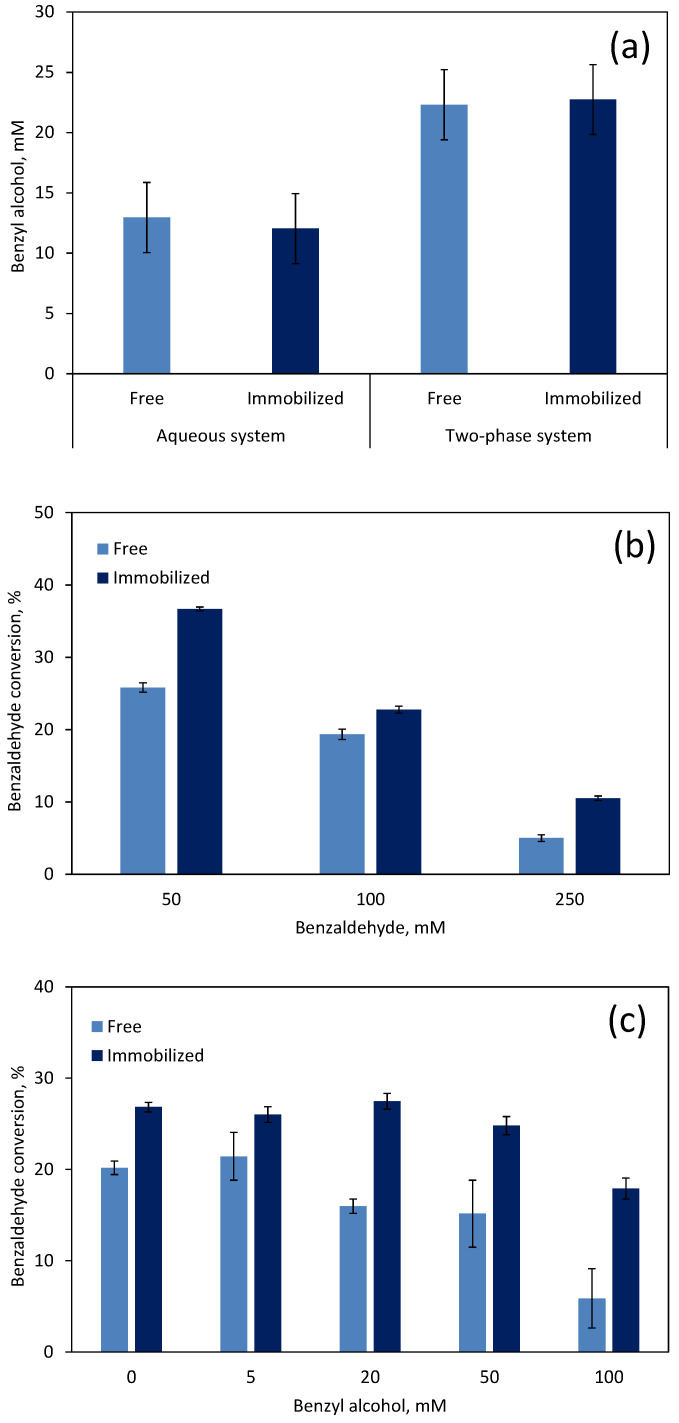
Effect of *G. arilaitensis* 232 cell immobilization on benzaldehyde conversion. (**a**) Comparison between the aqueous and the two-phase systems. (**b**) Effect of the initial benzaldehyde concentration on benzaldehyde conversion in the two-phase system. (**c**) Effect of benzyl alcohol concentration on benzaldehyde conversion in the two-phase system. Results are the average of three independent bioconversions. Data are presented as mean ± standard deviation.

In the present study, the *G. arilaitensis* 232 cells produced nearly the same amount of benzyl alcohol when free in suspension and immobilized (Figure 4a). This occurred both in aqueous and two-phase systems. In the latter system, the cells produced 1.8-fold (*p* < 0.001) more benzyl alcohol than in the aqueous system. When the reaction was prolonged for 20 h, and higher initial concentrations of substrate were used, the immobilized cells converted between 1.2- and 2.1-fold more benzaldehyde than free cells (Figure 4b). The immobilization of the cells could decrease the inhibitory effect of benzaldehyde, but a clear decrease in benzaldehyde conversion could still be observed with increasing substrate concentrations. The conversion of benzaldehyde at 250 mM was only 28.7% of that observed with 50 mM of substrate. Furthermore, the product benzyl alcohol also inhibited the catalytic activity of the *G. arilaitensis* 232 cells (Figure 4c). Under product inhibitory conditions, immobilized cells also converted higher amounts of benzaldehyde than free cells: at 100 mM benzyl alcohol, free cells degraded 34.7% of the amount converted when no product was initially present but immobilized cells converted 66.8%. The difference between the two forms of biocatalyst was more noticeable at 100 mM with immobilized cells converting 3 times more benzaldehyde. The performance of immobilized *G. arilaitensis* 232 cells was significantly affected at concentrations ca. 50 mM of benzyl alcohol, whereas free cells were inhibited above ca. 20 mM of product.

### 3.3. Scaling up of the Reaction System

**Figure 5:**
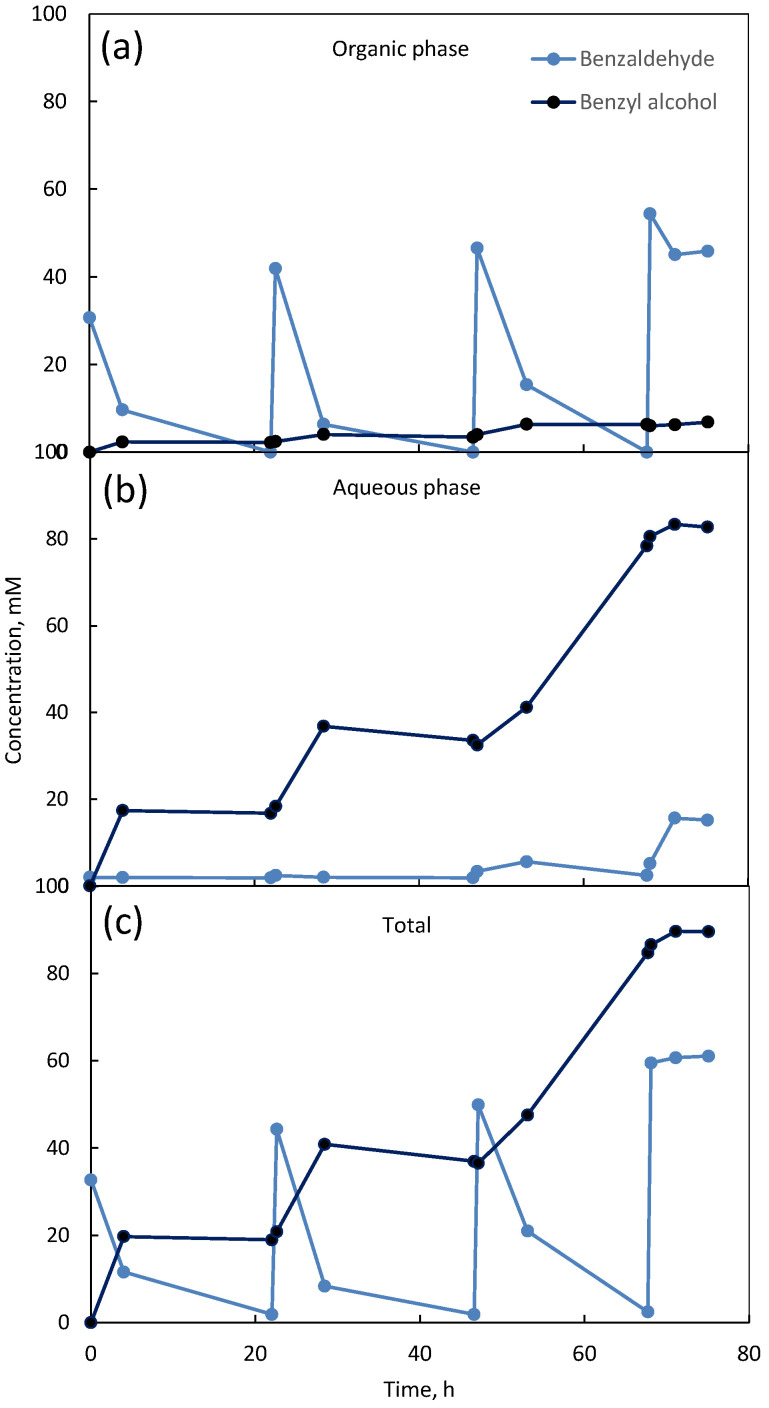
Two-phase stirred reactor system with 50 mL of total reaction volume. Benzaldehyde and benzyl alcohol concentrations over time in the organic phase (**a**), aqueous phase (**b**), and total concentration of both compounds in the system (**c**).

The information collected from the assays previously shown and discussed was used for the scaling up of the biocatalytic system. In the first case, the two-phase system was scale-up to a 50 mL reactor operated in fed-batch mode. This mode of operation allows substrate inhibition to be overcome since the concentration of substrate may be maintained below a given threshold [70]. The system started with a concentration of 50 mM of benzaldehyde. Three substrate additions equal to the initial concentration of substrate were made to the reactor once it was consumed (Figure 5). The benzaldehyde concentration was higher in the organic phase (Figure 5a) and the benzyl alcohol concentration was higher in the aqueous phase (Figure 5b). The conversion of benzaldehyde and consequent increasing benzyl alcohol in the system were observed (Figure 5c). However, after the last addition of substrate at 68 h, benzaldehyde was not converted. The maximum concentration measured of benzyl alcohol at the end of the experiment was 89.6 mM. In terms of mass balance, the product should be equivalent to the addition of the benzaldehyde consumed (150 mM in total). The explanation for the difference may be the evaporation of benzaldehyde or its absorption by the cells during reactor operation as observed in another study [58]. The fed-batch system was successful in enabling the conversion of 150 mM (accumulated value) of benzaldehyde by minimizing its inhibitory effect. The productivity achieved with the two-phase system was 0.122 g_benzyl alcohol_/g_DCW_Lh. However, the accumulation of the product in the aqueous phase probably ended the bioconversion. Benzyl alcohol concentrations above 50 mM had a strong inhibitory effect on free cells as discussed before (Figure 4c). Implementation of an in situ product removal system for the removal of benzyl alcohol could be used to improve the reaction and for simplification of the downstream process to obtain the purified product [71].

In the second case studied, the system was scaled-up to a continuous plug flow reactor (CPFR). Continuous flow biocatalysis is a big area of study with proven diversity of applications in different industries [72]. In the present study, the aqueous medium containing benzaldehyde passed through the column packed with immobilized cells (Figure S3). The conversion is thus dependent on the length of the column: the concentration of product increases along the column which the substrate decreases. The residence time of the CPFR, 1.5h, was adjusted to the activity of the immobilized cells and more than 99% of benzaldehyde conversion was achieved at the end of the column (Figure S4). The maximum calculated productivity was 1.16 g_benzyl alcohol_/g_DCW_Lh.

Compared to the fed-batch system, the CPFR represents a 10-fold (*p* < 0.02) improvement in productivity. The results support the known advantage of higher volumetric productivity of CPBR over stirred reactor systems [73]. Moreover, the CPBR allows a better separation of the biocatalyst from the product, thus simplifying downstream processing. Another important aspect to take into consideration is the operating lifetime of the biocatalyst. This is important for commercial implementation of biocatalytic systems because of its contribution to the final cost of the product [73]. A successful industrial implementation requires hundreds of recycles per biocatalyst [27]. The immobilized *G. arilaitensis* 232 cells used showed good stability over five cycles of benzaldehyde conversion (Figure S5). The results indicate good stability and potentially good operating lifetime of the biocatalyst but further experiments are required.

The CPFR studied in the present work is a valid sustainable alternative to the current chemical reaction processes used for benzyl alcohol production. Continuous-flow mode minimizes waste generation, reducing the *E* factor [13]. The origin of benzaldehyde is crucial to achieve a complete sustainable process. Benzaldehyde from agro-industrial waste, such as apricot juice, or from wastewaters produced by pharmaceutical and cosmetic industries, could be used. These feedstock sources may contribute to a circular economy. The migration from a linear to a circular economy design is essential to develop greener and sound biochemical processes.

## 4. Conclusions

The implementation of a two-phase system, with hexane as organic phase, allowed a higher load capacity and conversion of benzaldehyde when compared to a single aqueous system. The scaling up of the system to a fed-batch stirred bioreactor allowed the conversion of 150 mM of benzaldehyde. However, product accumulation halted the reaction. A continuous plug flow reactor with immobilized cells allowed a 10-fold increase in productivity in comparison with the fed-batch stirred reactor. Our study demonstrates the capacity of marine bacteria for the production of a valuable flavor compound, and makes a significant contribution to the development of sustainable processes for benzyl alcohol production.

## Data Availability

The data presented in this study are available on request from the corresponding author. The data are not publicly available due to confidentiality agreements.

## Supplementary Materials

The following supporting information can be downloaded at: https://www.mdpi.com/article/10.3390/microorganisms10050966/s1, Scheme S1: Conversion of benzaldehyde to benzyl alcohol by *Glutamicibacter arilaitensis* whole cells; Figure S1: Determination of the optimal temperature and pH for the conversion of benzaldehyde by *Glutamicibacter arilaitensis* cells; Figure S2: Effect of the initial concentration of benzaldehyde on benzaldehyde conversion by *G. arilaitensis* 232 cells in a two-phase system using n-hexane as organic phase after 20 and 40 h; Figure S3: Continuous plug flow reactor (CPFR) setup for the conversion of benzaldehyde to benzyl alcohol; Figure S4: TIC chromatograms obtained by GC-MS of the acetyl acetate used to extract substrate and product; Figure S5: Relative conversion at sequentially cycles of 1 h conducted in 10 mL Verex^TM^ Headspace vials.
