# Supplementary material for: Process Development for Benzyl Alcohol Production by Whole-Cell Biocatalysis in Stirred and Packed Bed Reactors
Source: Microorganisms. 2022 May 3;10(5):966. doi: 10.3390/microorganisms10050966 (PMC9147996; doi:10.3390/microorganisms10050966)
Supplement: Supplementary file 1 [file microorganisms-10-00966-s001.zip › microorganisms-1695383-supplementary.pdf]

Supplementary Material

Process development for benzyl alcohol production by whole-cell biocatalysis in stirred and packed bed reactors

Carlos J. C. Rodrigues[1,2] and Carla C C. R. de Carvalho[1,2*]

[1] iBB-Institute for Bioengineering and Biosciences, Department of Bioengineering, Instituto Superior Técnico, Universidade de Lisboa, Av. Rovisco Pais, 1049-001 Lisbon, Portugal; carlos.junio@tecnico.ulisboa.pt
[2] Associate Laboratory i4HB—Institute for Health and Bioeconomy, Instituto Superior Técnico, Universidade de Lisboa, Av. Rovisco Pais, 1049-001 Lisbon, Portugal
[*] Correspondence: ccarvalho@tecnico.ulisboa; Tel.: +351 21 841 9594

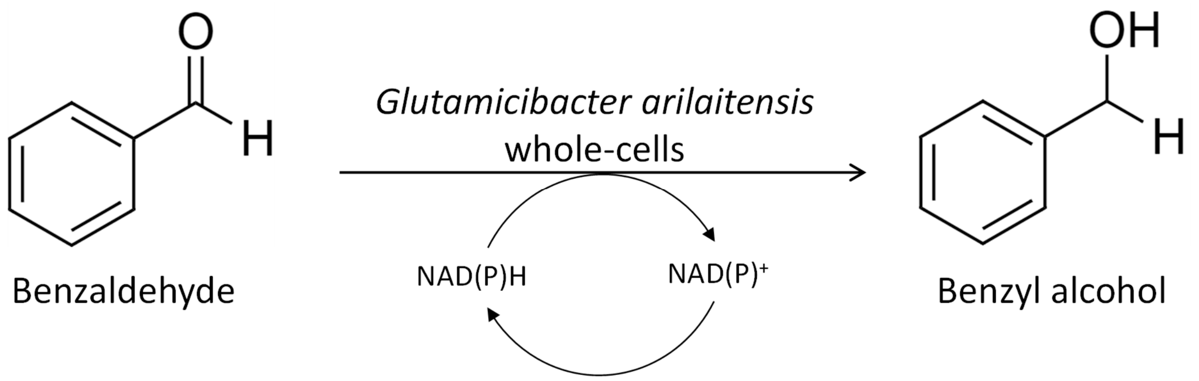

Scheme S1. Conversion of benzaldehyde to benzyl alcohol by *Glutamicibacter arilaitensis* whole cells.

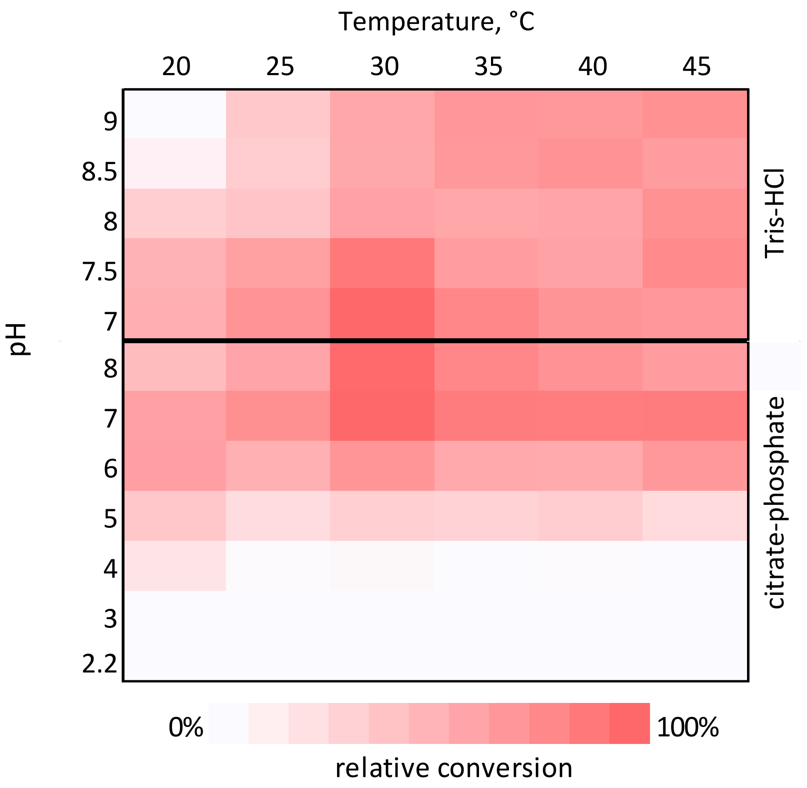

Figure S1. Determination of the optimal temperature and pH for the conversion of benzaldehyde by *Glutamicibacter arilaitensis* 232 cells. The optimal temperature and pH for the conversion of benzaldehyde to benzyl alcohol was determined using arrays of temperature and pH conditions. For the pH range, two different buffers were used: citrate-phosphate buffer was used from pH 2.2 to 7; and the Tris-HCl 100 mM buffer was used from pH 7 to 9. The reactions were conducted with the respective buffer in 1 mL volume containing: 10% (v/v) DMSO, 20 mM benzaldehyde and 10 mg DCW /mL. The reactions were stopped after 2 h and extracted with ethyl acetate for analytical determination of benzaldehyde and benzyl alcohol by GC-MS. Reactions were performed in triplicate at 30°C in 10 mL Verex™ Headspace vials closed with screwed Verex Caps with bonded-in PTFE/silicone septa (Phenomenex, USA), with magnetic agitation set at 800 rpm (using 12mm×3mm stirrer bar from Kartell, New York, NY, USA).

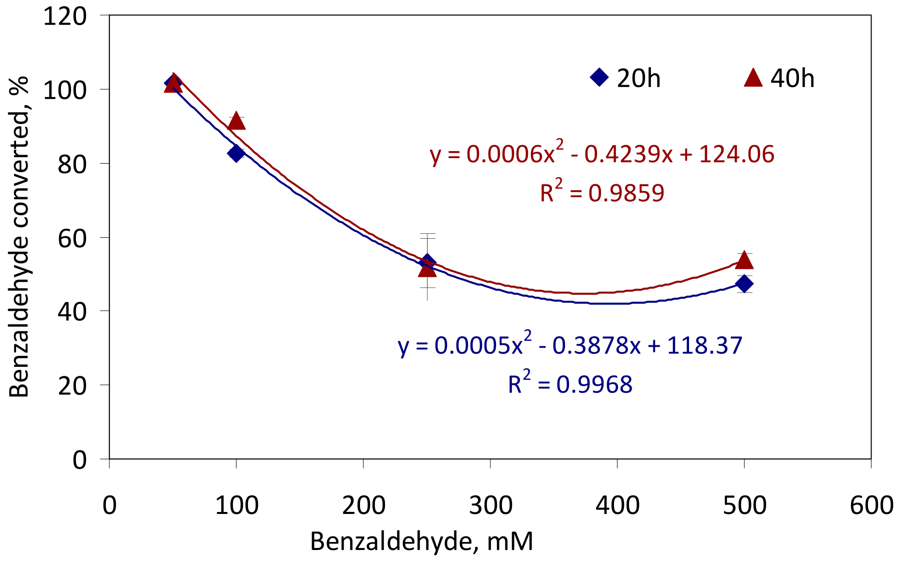

Figure S2. Effect of the initial concentration of benzaldehyde on benzaldehyde conversion by *G. arilaitensis* 232 cells in a two phase system using *n*-hexane as organic phase after 20 and 40h. Data points adjusted to second degree polynomial trendlines.

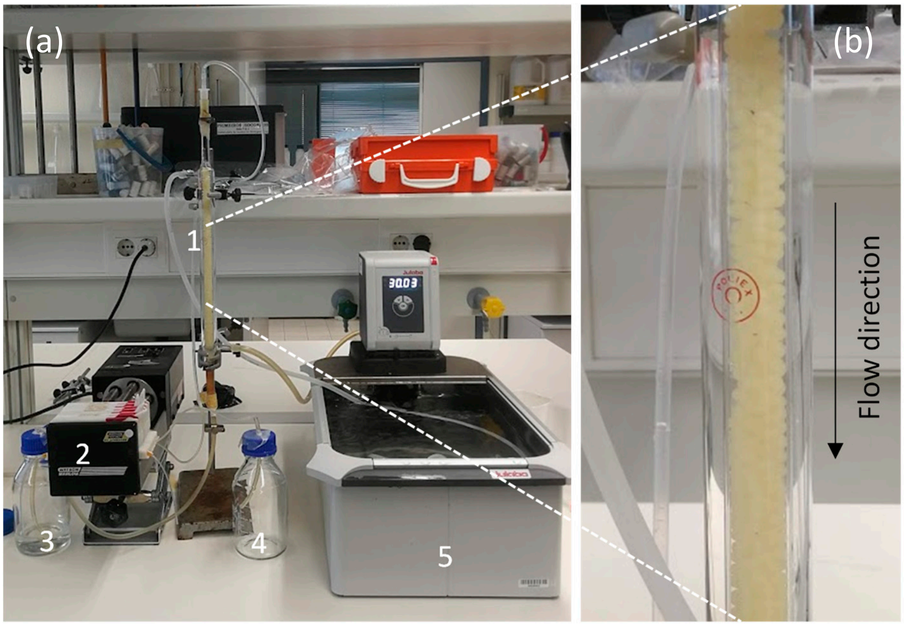

Figure S3. Continuous plug flow reactor (CPFR) setup for the conversion of benzaldehyde to benzyl alcohol using *G. arilaitensis* 232 cells immobilized in sodium alginate (a) and detailed image of the biocatalyst inside the CPFR (b). 1 – jacked glass column packed with the biocatalyst (volume of 25 mL when empty), 2 – peristaltic pump, 3 – inlet reservoir with medium containing 15 mM of benzaldehyde, 4 – outlet reservoir, 5 – water bath with recirculation through the jacked column to maintain the reaction temperature at 30°C. The column was packed with 19.2 g of immobilized *G. arilaitensis* 232 cells in calcium alginate beads corresponding to 273.55 mg_{DCW}. The volume of void (liquid surrounding the alginate beads) was 7 mL. The measured residence time was 1.5 h.

(a)

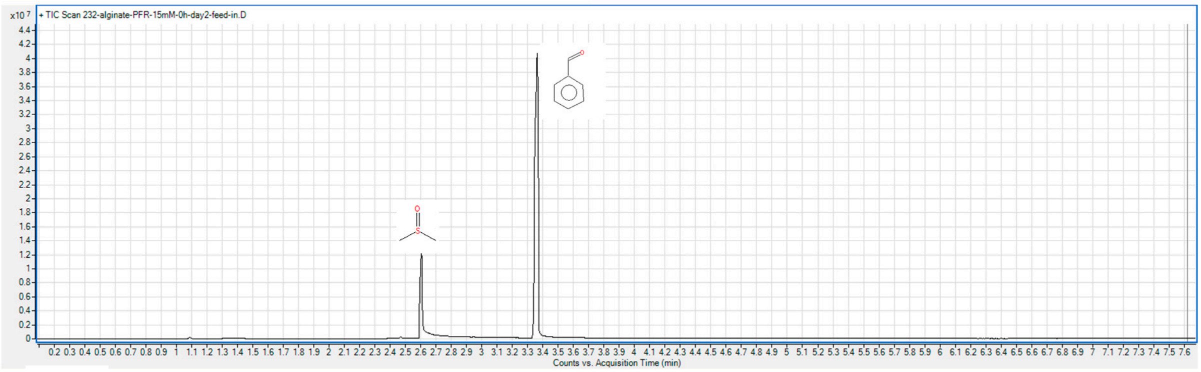

(b)

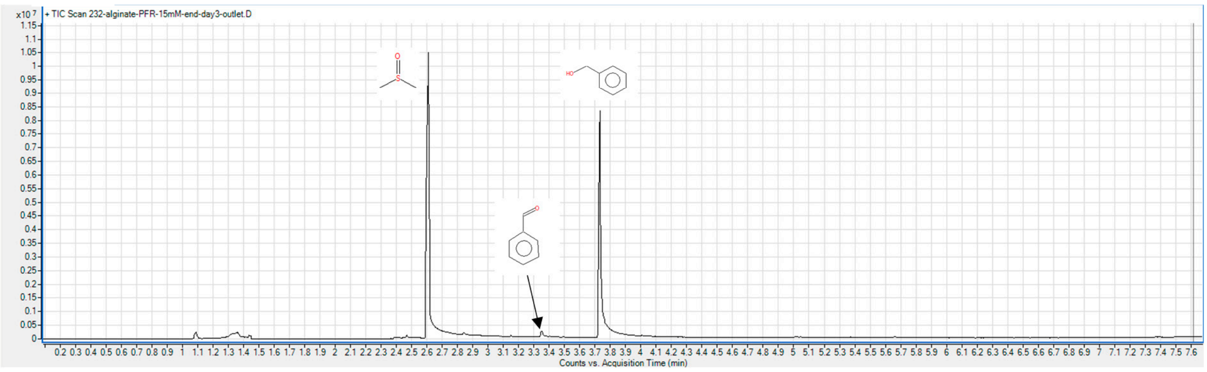

Figure S4. TIC chromatograms obtained by GC-MS of the acetyl acetate used to extract substrate and product from the inlet (a) and outlet reaction medium (b) of the CPFR after several hours of operation. DMSO, RT = 2.61 min; Benzaldehyde, RT= 3.35 min; Benzyl alcohol, RT = 3.73 min.

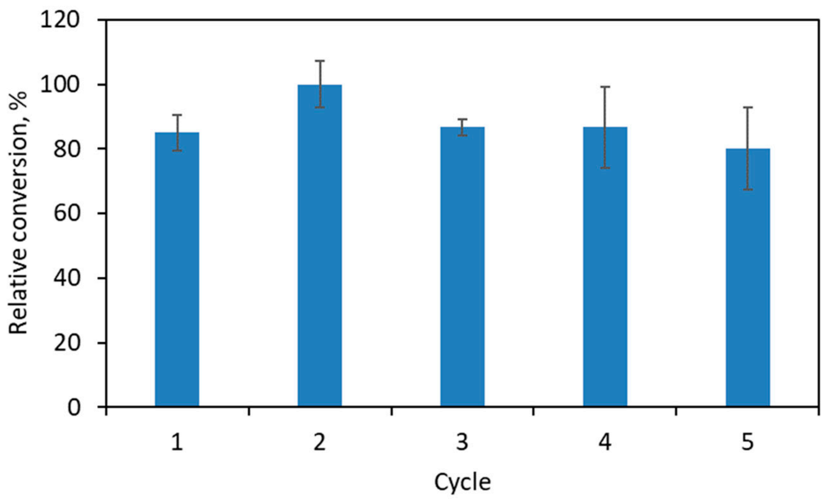

Figure S5. Relative conversion at sequentially cycles of 1h conducted in 10 mL Verex™ Headspace vials closed with screwed Verex Caps with bonded-in PTFE/silicone septa (Phenomenex, Torrance, CA, USA). The 1 mL reaction media contained: 5 mM of benzaldehyde, 5% (v/v) DMSO, Tris-HCl 100 mM pH 7.5 and 10 mg of immobilized *G. arilaitensis* 232 cells in sodium alginate. The reaction was carried out at 30°C with magnetic agitation (400 rpm) for mixing. The product concentration at the end of each cycle was used to determine the relative activity.
